# Supplementary material for: Chronotype changes after sex hormone use: A prospective cohort study in transgender users of gender-affirming hormones
Source: Chronobiol Int. 2024 Apr 14;41(5):658–68. doi: 10.1080/07420528.2024.2339989 (PMC11132553; doi:10.1080/07420528.2024.2339989)
Supplement: Supplemental Material [file ICBI_A_2339989_SM3414.pdf]

Supplementary materials

1. Complete case analysis

Supplementary Table 1 below shows the outcomes of the main analyses, conducted in only participants who contributed both a baseline and a follow-up measurement (i.e., complete case analysis). Outcomes of these analyses indicate that there is no notable difference between the overall analyses and the complete case analyses.

Supplementary Table 1. Complete case analysis of sleep duration and (sleep-corrected) Midpoint of Sleep on Free days (MSF_{sc}) obtained from the µMCTQ in the transmasculine (TM) group and the transfeminine (TF) group. All variables are reported in clock hours (hh:mm).

**Outcome** *in hh:mm*	***Transmasculine group***			
	Predictor (n)	*Mean (SD)*	**Unadjusted model** *Estimated change from baseline (95% CI: L; H) p-value)*	**Adjusted model** *Estimated change from baseline (95% CI: L; H) p-value)*
**MSF_{sc}**	Baseline (37)	04:35 (01:31)	Reference	Reference
	3-month follow up (37)	04:55 (01:38)	00:20 (-00:03; 00:42) p = 0.09	00:24 (00:02; 00:46) p = 0.037
**Sleep duration**	Baseline (37)	08:30 (01:19)	Reference	Reference
	3-month follow up (37)	08:43 (01:05)	00:12 (-00:10, 00:34) p = 0.29	00:12 (-00:10, 00:35) p = 0.30
Outcome in hh:mm	***Transfeminine group***			
	Time point, months after start GAHT (n)	*Mean (SD) or median (IQR)* [1.]	**Unadjusted model** *Estimated change from baseline (95% CI: L; H) p-value)*	**Adjusted model** *Estimated change from baseline (95% CI: L; H) p-value)*
**MSF_{sc}**	Baseline (37)	04:54 (01:20)	Reference	Reference
	3-month follow up (37)	04:39 (01:02)	-00:14 (-00:35; 00:06) p = 0.17	-00:20 (-00:37; -00:02) p = 0.031
**Sleep duration**	Baseline (37)	08:22 (01:03)	Reference	Reference
	3-month follow up (37)	08:25 (00:54)	00:03 (-00:18; 00:23) p = 0.79	-00:01 (-00:20; 00:19) p = 0.95

2. Role of cycle regulation use

We conducted an additional sensitivity analysis with a covariate for cycle regulation use (i.e., use of progestins or hormonal contraceptives), and results are displayed in Supplementary Table 2 below. Results of this analysis indicate that use of cycle regulation is associated with an earlier chronotype.

Supplementary Table 2. Adjusted analyses incorporating cycle regulation use in the transmasculine (TM) group. All variables are reported in clock hours (hh:mm).

Outcome *in hh:mm*	***Transmasculine group – No covariate for work status***		
	Predictor (n)	***3 months of GAHT vs. baseline*** *Estimated change from baseline (95% CI: L; H) p-value)*	***Use of cycle regulation vs. no use of cycle regulation*** *Estimated change from baseline (95% CI: L; H) p-value)*
MSF$_{sc}$	Baseline (37)	Reference	Reference
	3-month follow up (37)	00:12 (-00:11; 00:35) p = 0.31	
	Cycle regulation use (29)		-00:35 (-01:08; -00:01) p = 0.050
Sleep duration	Baseline (37)	Reference	Reference
	3-month follow up (37)	00:17 (-00:06, 00:39) p = 0.15	
	Cycle regulation use (n)		00:15 (-00:16, 00:45) p = 0.34
Outcome *in hh:mm*	***Transmasculine group – Added covariate for work status***		
	Predictor (n)	***3 months of GAHT vs. baseline*** *Estimated change from baseline (95% CI: L; H) p-value)*	***Use of cycle regulation vs. no use of cycle regulation*** *Estimated change from baseline (95% CI: L; H) p-value)*
MSF$_{sc}$	Baseline (37)	Reference	Reference
	3-month follow up (37)	00:17 (-00:05; 00:39) p = 0.15	
	Cycle regulation use (n)		-00:32 (01:05; 00:01) p = 0.061
Sleep duration	Baseline (37)	Reference	Reference
	3-month follow up (37)	00:17 (-00:05, 00:41) p = 0.14	
	Cycle regulation use (n)		00:15 (-00:15, 00:46) p = 0.33
